# Adaptive Waveform Design for MIMO Radar-Communication Transceiver

**DOI:** 10.3390/s18061957

**Published:** 2018-06-16

**Authors:** Yu Yao, Junhui Zhao, Lenan Wu

**Affiliations:** 1 School of Information Engineering, East China Jiaotong University, Nanchang 330031, China; jhzhao@ecjtu.edu.cn; 2 School of Information Science and Engineering, Southeast University, Nanjing 210096, China; wuln@seu.edu.cn

**Keywords:** multiple input multiple output (MIMO), joint radar-communication, relative entropy (RE), mutual information (MI), waveform optimization

## Abstract

The system architecture for an adaptive multiple input multiple output (MIMO) radar-communication transceiver is proposed. A waveform design approach for communication data embedding into MIMO radar pulse using M-ary position phase shift keying (MPPSK) waveforms is introduced. A waveform optimization algorithm for the adaptive system is presented. The algorithm aims to improve the target detection performance by maximizing the relative entropy (RE) between the distributions under existence and absence of the target, and minimizing the mutual information (MI) between the current received signals and the estimated signals in the next time instant. The proposed system adapts its MPPSK modulated inter-pulse duration to suit the time-varying environment. With subsequent iterations of the algorithm, simulation results show an improvement in target impulse response (TIR) estimation and target detection probability. Meanwhile, the system is able to transmit data of several Mbps with low symbol error rates.

## 1. Introduction

### 1.1. Joint Radar-Communications Transceivers

Radars with multiple functions have attracted substantial interest in recent years and sparked a number of research initiatives [1,2,3,4]. It is studied in [5] that the intelligent transportation system (ITS) employs communication devices to convey traffic information and utilizes the radar device to sense the traffic circumstances, which motivates the integration of radar and communications.

The objective of the joint design is to increase both the energy efficiency and the spectrum efficiency, and to reduce manufacturing cost as well. The integrated transceiver designs can be classified into two main categories. One category is based on the multiplexing technique, including space division multiplexing, time division multiplexing (TDM), frequency division multiplexing (FDM), and code division multiplexing [6,7]. However, these kinds of approaches have a common defect in that target detection and digital communication cannot be operated simultaneously in some domains. For instance, the radar and communication cannot operate in the same time slot for the methods based on the TDM technique. The other category is based on waveform sharing, and consists of two types: (i) the information is hidden in the conventional radar waveforms; (ii) the communication waveforms are either slightly changed or not. Aubry considered waveform design in a spectrally crowded environment where some frequency bands are shared among the radar and communication system [8]. References [9,10] considered the synthesis of waveforms optimizing radar performance while satisfying multiple spectral compatibility constraints. A suitable modulation technique of the transmitted waveform energy was developed in [11], which achieves an enhanced spectral coexistence with the surrounding electromagnetic environment. Spectrum sharing between multiple input multiple output (MIMO) radars and communication system was initially considered in [12], where the radar interference to the communication system was eliminated by projecting the radar waveforms onto the null space of the interference channel. However, projection-type techniques might miss targets lying in the row space of the interference channel. Furthermore, the interference generated by the communication system to the radar was not considered in [13].

### 1.2. Adaptive MIMO Radar Waveform

Cognitive radar (CR) systems can adjust their transmission waveform and receive filters adaptively based on the prior knowledge of targets and the environment, thus the have potential in enhancing the detection and recognition performance for targets [14]. In CR, cognition plays a critical role in the feedback loop, which includes long-term memory. For example, geographic map and elevation models, and short-term memory developed by the receiver online. Aubry considered the problem of knowledge-aided and cognitive constant envelope signal transmission and receive filters’ joint optimization in a signal-dependent clutter environment [15,16]. A cognitive approach to design phase-only modulated waveforms sharing a desired range Doppler response was proposed [17]. The idea is to minimize the average value of the ambiguity function of the transmitted signal over some range Doppler bins, which are identified exploiting a plurality of knowledge sources. To deal with the general signal-dependent interfering scenario, the joint design of the transmission signal and the receive filter bank for a radar system operating in the presence of possible range-ambiguous signal-dependent disturbances was discussed in [18]. The problem of joint transmission code and receiver filter design was considered to optimize the achieved SINR of extended targets [19]. Robust joint optimization of the transmitted signal and receive filter bank for the extended target and polarimetric radar in the presence of signal-dependent and signal-independent interference was considered in [20].

Compared with the traditional phased array radar, which transmits a scaled version of a single waveform, MIMO radar offers enhanced capabilities [21] through waveform diversity and has drawn considerable attention in recent years. Waveform design for MIMO radar has been intensively investigated [22,23,24]. Spatial beampatterns ranging from highly directional beams to omnidirectional beams can be achieved through various adaptive transmission methods [25]. Some of the noteworthy works in this area include [26,27,28], where the radar transmission parameters are continuously modified in order to improve the target parameter estimation in a time-varying radar environment. The related works on designing estimation waveforms for MIMO radar systems include [29], which discussed the equivalence between maximizing MI and minimizing the mean square estimation error (MSE). An algorithm for optimal waveform design based on maximizing the output signal-to-plus-noise ratio (SNR) and the mutual information (MI) between the target ensemble and observations was derived in [30]. In [31] it was shown that maximizing the MI between the target impulse response (TIR) and the observations may enable the radar system a better capability in characterizing a target in noisy environments. Yang and Blum [32] extended the work in [30] by using the MI between the random target response and the reflected signal as a waveform optimization criterion in the MIMO radar configuration. In [33] space-time code optimization for MIMO radar based on MI was considered. Other existing works [34,35,36,37] also utilize similar design criteria.

### 1.3. Adaptive MIMO Radar-Communication Waveform Design

In this paper, we analyze the performance of a MIMO radar-communication transceiver that contains the concept of “cognitive” and “joint radar-communication”. We propose a composite waveform design scheme for M-ary position phase shift keying (MPPSK) modulated communication symbols embedded in a MIMO radar pulse. The principle of the proposed strategy is embedding MPPSK symbols by phase-rotating the transmit waveforms of the MIMO radar. The phase-rotation is transparent to the radar operation and does not compromise the offerings of the radar functionality. We present an optimization algorithm for the proposed composite waveform, which is summarized as follows:

(1) We design transmission waveforms by maximizing the relative entropy (RE) between the distributions with and without targets, subjecting to the transmission power constraint. The optimization waveform should “match” the target and noise.
(2) We formulate the criterion of waveform selection based on minimizing MI between successive radar echoes, with an intention of estimating the target parameters.

The main contributions of this paper are summarized as follows:

(1) We develop a new scheme for communication data embedding into adaptive MIMO radar;
(2) We present a novel framework for an adaptive MIMO radar-communication system, which benefits from the principle of cognition radar;
(3) We design a novel algorithm for waveform optimization in the adaptive distributed MIMO radar-communication framework;
(4) We provide performance analysis of the MIMO radar-communication system network in terms of receiver operating characteristics (ROC), detection probabilities and communication symbol error rates (SER) between the proposed systems.

The paper is organized as follows: in Section 2, an adaptive MIMO radar-communication system architecture is described. In Section 3, we present a MIMO radar signal model and MPPSK information embedding scheme. In Section 4, we formulate a novel waveform optimization algorithm. The transmitted waveforms are designed based on RE and selected based on the MI criterion. The simulation results illustrating the proposed methods are provided in Section 5, and concluding conclusions are drawn in Section 6.

Throughout this paper, the following notations will be used. Vectors are denoted by boldface lowercase letters and matrices by boldface uppercase letters. H and Re(⋅) denote transpose conjugate operation and the real part of a variable, respectively. ${\left\{\cdot \right\}}^{T}$ stands for the transpose operation and ${\left\{\cdot \right\}}^{H}$ denotes the Hermitian transpose. det{⋅} denotes the determinant of a matrix. The conjugate denoted as ${\left(\cdot \right)}^{*}$, linear convolution operator as ∗, expectation operator as E{⋅} and variance operator as Var{⋅}. diag{⋅} denotes a diagonal matrix with the indicated entries.

## 2. System Architecture and Network

A set of orthogonal waveforms is used to implement the primary MIMO radar operation. The secondary communication function is implemented by embedding one MPPSK communication symbol in each orthogonal waveform, i.e., the number of embedded communication symbols during each radar pulse equals the number of transmit antennas (described in Section 3). The data link can be established between two nodes.

**Figure 1:**
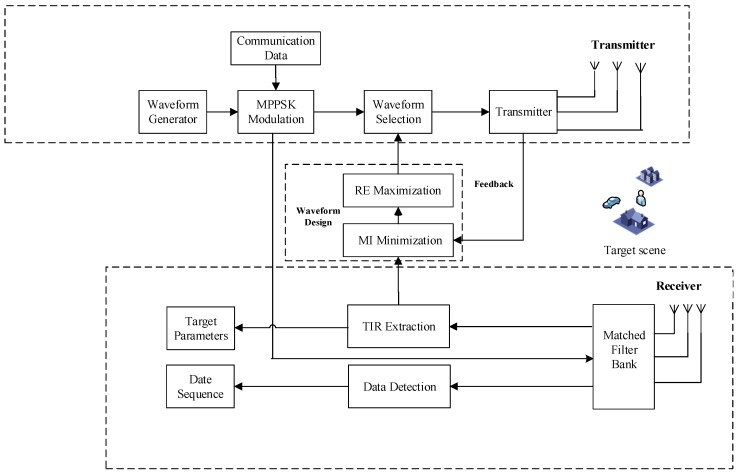
The system architecture of adaptive MIMO radar-communication transceiver.

The received signals are processed by a matched filter bank, which matches the signals to each individual transmission waveform stored in the receiver. The communication receiver would perform the matched filters to undo the phase shift from the received signals. Consequently, the embedded symbols are estimated. The receiver compares the estimates to the dictionary to find the embedded communication symbols and convert them into the corresponding transmission sequence. The RE and MI module designs and selects a suitable waveform for the transmitter to acquire the best knowledge about the target in the next time instant (described in Section 4). Figure 1 describes the system architecture of the adaptive MIMO radar-communication transceiver.

**Figure 2:**
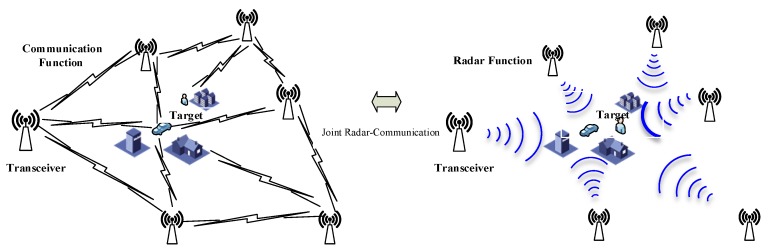
The joint communication-radar operation.

In order to simplify the discussion, we assume perfect synchronization between different adaptive MIMO radar-communication transceivers. Figure 2 shows the joint communication-radar operation.

## 3. MIMO Radar Signal Model and Information Embedding Scheme

We consider that a distributed MIMO radar system equipped with a transmit array and a receive array comprising *M* antennas and *N* antennas. The transmitting and receiving antennas are arranged in an arbitrary linear array. The minimum antenna spacing is larger than half wavelength. Two different antenna elements are independent. In this paper, intelligent transportation system (ITS) applications scenario is considered, that is, the round trip distance of transmitted waveform via the target is no more than tens of meters. We consider the scenario that the length of the transmitted waveform is much larger than the maximum delay with respect to the first arrival among all the links.

**Figure 3:**
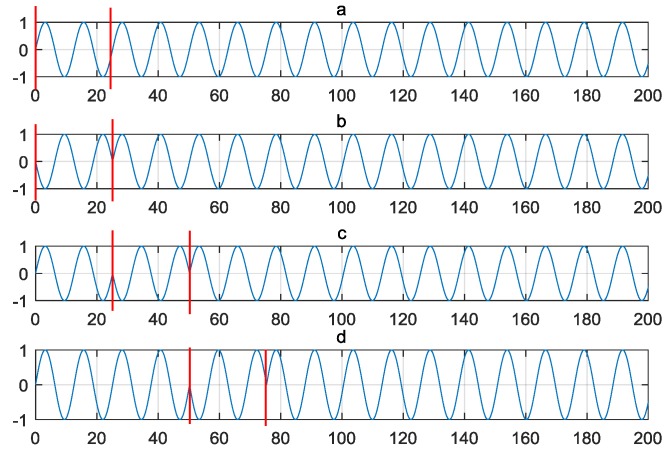
(**a**) 4-PPSK modulated waveform for symbol “0”; (**b**) 4-PPSK modulated waveform for symbol “1”; (**c**) 4-PPSK modulated waveform for symbol “2”; (**d**) 4-PPSK modulated waveform for symbol “3”.

We utilized a set of orthonormal waveforms $s_{m}\left(t\right),m=1,\ldots ,M$ for transmission, that is, $\int_{T_{P}}s_{m}\left(t\right)s_{k}^{*}\left(t-\tau \right)dt=0$ for all k≠m, $\int_{T_{P}}{\left|s_{m}\left(t\right)\right|}^{2}dt=1$. $T_{p}$ denotes the waveform duration. $s\left(t\right)={\left[s_{1}\left(t\right),\ldots ,s_{M}\left(t\right)\right]}^{T}$ indicates the M×1 vector of orthogonal waveforms. Then, we present the MIMO radar system with MPPSK information embedding scheme. MPPSK modulated waveforms are defined as follows:

(1) $$g\left(t\right)=\{\begin{array}{cc} g_{0}(t)=\sin2\pi f_{c}t, & 0\leq t<NT_{c}\\ g_{p}\left(t\right)=\{\begin{array}{c} \sin(2\pi f_{c}t)\\ -\sin(2\pi f_{c}t)\\ \sin(2\pi f_{c}t) \end{array} & \begin{array}{cc} & \begin{array}{c} \begin{array}{l} 0\leq t\leq (p-1)KT_{c}\\ (p-1)KT_{c}<t<pKT_{c} \end{array}\\ KT_{c}\leq t<NT_{c} \end{array} \end{array};1\leq p\leq P-1 \end{array}$$

with $g_{0}(t)$ and $g_{p}\left(t\right)$ being modulation waveforms of symbol “0” and “p(p>0)”, $f_{c}$ and $T_{c}$ represent the carrier frequency and the carrier period, respectively. K and N are modulation parameters, which denote the number of the carrier period in each time slot and the number of the carrier period in each symbol, respectively. p(p=0,1,…,P−1) is M-ary source symbol. Hence, increased P leads to higher data rate as more time slots are utilized. The waveforms of 4-PPSK modulation are illustrated as in Figure 3. The coefficient for the x axis is the index of a certain sample point. Setting P=4; K=2; N=20.

The modulation waveform for symbol “0” is a sinusoidal as shown in Figure 3a, Figure 3b illustrates the modulation waveform for symbol “1” with the phase hopping during the first two carrier period (from 0 to 20), the next (from 20 to 40) is for symbol “2” in Figure 3c, and last (from 40 to 60) is for symbol “3” in Figure 3d. The MPPSK modulated signal has the capability of high precise ranging measurement. The time hopping scheme for MPPSK waveform has been analyzed in [37].

During each radar pulse, M of MPPSK symbols can be embedded into the MIMO radar emission. Thus, during the *i*-th pulse, the phase symbol $\Omega_{M}\left(i\right)\in {\mathbb{R}}_{MPPSK},\;m=1,\ldots ,M$ can be selected from a predefined a dictionary of $T=P^{B}$ symbols. We assume the dictionary is uniformly distributed within the interval [0,2π], that is, ${\mathbb{R}}_{MPPSK}=\left\{\begin{array}{cccc} 0 & \frac{2\pi }{T}, & \ldots , & \frac{(T-1)2\pi }{T} \end{array}\right\}$. During the *i*-th pulse, the phase rotated set of transmitted waveforms can be denoted as:

(2) x(t,i)=Π(i)s(t)

where:

(3) $$\Pi \left(i\right)=diag\left\{\left[\begin{array}{ccc} e^{j\Omega_{1}\left(i\right)}, & \ldots & e^{j\Omega_{M}\left(i\right)} \end{array}\right]\right\}$$

We further consider that a single antenna communication receiver is located at an arbitrary direction $\theta_{0}$. Then, the received signals can be expressed as:

(4) $$y\left(t,i\right)=s^{T}\left(\theta_{0}\right)x\left(t,i\right)+n\left(t,i\right)$$

where $s^{T}\left(\theta_{0}\right)$ is the steering vector of the transmitting array in direction $\theta_{0}$. n(t,i) is additive white Gaussian noise (AWGN) with zero mean and variance $\delta_{w}^{2}$. Matched-filtering y(t,i) to $s_{m}\left(t\right),m=1,\ldots ,M$ yields:

(5) $$\begin{array}{ll} y_{m}\left(i\right) & =\int_{T_{p}}y\left(t,i\right)s_{m}^{*}\left(t\right)dt\\ & =\int_{T_{p}}s^{T}\left(\theta_{0}\right)x\left(t,i\right)s_{m}^{*}\left(t\right)dt\\ & =\int_{T_{p}}s^{T}\left(\theta_{0}\right)\Pi \left(i\right)s(t)s_{m}^{*}\left(t\right)dt\\ & =s_{m}^{\prime }e^{\Omega_{m}\left(i\right)}+n_{m}\left(i\right),m=1,\ldots ,M \end{array}$$

where $s_{m}^{\prime }≜e^{-j2\pi r_{m}\sin\theta_{0}}$ is the *m*-th entry of $s^{T}\left(\theta_{0}\right)$, $r_{m}$ is the displacement between the first and the *m*-th elements of the transmit array measured in wavelength, and $n_{m}\left(i\right)$ is the AWGN with zero mean and variance $\delta_{n}^{2}$. Hence, the received communication signal at the output of the *m*-th matched filter is a phase-shifted and noisy term of the *m*-th entry of $s\left(\theta_{0}\right)$, meaning that the phase shift $\Omega_{m}\left(i\right),m=1,\ldots ,M$ can be recovered from $y_{m}\left(i\right),m=1,\ldots ,M$. The embedded phase symbols can be estimated as follows:

(6) $$\Omega_{m}\left(i\right)≜angle\left(y_{m}\left(i\right)\right)+2\pi r_{m}\sin\theta_{0}$$

where angle(.) denotes the angle of a complex number. The receiver has complete knowledge of the displacement of the transmit array elements from the reference element. Thus, the receiver is ability to cancel the phase term. Once the embedded phase $\Omega_{m}\left(i\right)$ is estimated, the communication receiver compares the estimates to the dictionary ${\mathbb{R}}_{MPPSK}$ to find the MPPSK embedding symbols and convert them into the corresponding M-ary sequence.

We consider each MPPSK symbol represents B bits of M-ary information. The data rate of the proposed integrated system can be achieved as follows:

(7) R=B·M·PRF

where M denotes the number of transmit antennas. In radar applications with a high pulse repetition frequency (PRF), for example, millimeter-wave radar, a data rate in the tens of Mbps can be obtained.

## 4. (Two-Step) Waveform Optimization

During the *i*-th pulse, the phase rotated set of MPPSK embedding waveforms x(t,i) can be presented as a matrix $X_{i}\in {\mathbb{C}}^{M\times M}$ after discrete sampling. Here ℂ indicates the complex number domain. We assume $H_{i}={\left[h_{m,n}^{i}\right]}_{M\times N}$ and $N=\left[\begin{array}{cccc} n_{1} & n_{2} & \ldots & n_{N} \end{array}\right]$ are the TIR matrix and AWGN matrix, respectively. $h_{m,n}$ represents the TIR between the *m*-th transmit antenna and the n-th receive antenna. At the result, the M×N matric of the received signals can be expressed as:

(8) $$Y_{i}=X_{i}H_{i}+N$$

where $H_{i}∼\mathbb{C}Ν\left(0,R_{H_{i}}\right)$ and $N∼\mathbb{C}Ν\left(0,R_{N}\right)$. $R_{H}=E\left\{H_{i}^{H}H_{i}\right\}$ and $R_{N}=E\left\{N^{H}N\right\}$ are the covariance matrices of the target response $H_{i}$ and the noise N, respectively.

### 4.1. Waveform Design Based on Relative Entropy

We present a novel optimization algorithm for adaptive MIMO radar waveform design:

*Step 1*: waveform optimization. The main objective is to maximize the RE between the distributions under no target and target exists, subject to the transmission power constraint. The optimal waveform should “match” with the target and noise. Once the optimization waveform ensemble is acquired, the next step is to choose the best possible waveforms for emission from the ensemble. 

*Step 2*: Waveform selection. We formulate the criterion of waveform selection based on minimizing MI between successive radar echoes. The successive radar echoes are statistically independent on each other in time, with an intention of gaining more target feature information at each time instant of reception.

Step 1: Waveform optimization. Maximization of RE between the distributions under no target and target exists at time i. We intend to maximize the RE. This implies that the backscattering signals are more statistically dependent upon the actual radar scene. The idea of RE for adaptive waveform design stems from Stein’s lemma [38], which is presented as follows:

**Theorem** **1:** *Assume a binary hypothesis testing problem between the alternatives*$H_{0}$*and*$H_{1}$*Two distributions*$p_{0}$*and*$p_{1}$*is under the alternatives*$H_{0}$*and*$H_{1}$, *respectively. The RE between*$p_{0}$*and*$p_{1}$*is expressed as*

(9) $$D\left(p_{0}∥p_{1}\right)=\int p_{0}\log p_{0}|p_{1}$$

$A_{n}$ and $A_{n}^{c}$ are two acceptance regions for hypothesis $H_{0}$ and $H_{1}$. Let the error probabilities of the two types be $\alpha_{n}=p_{0}^{n}\left(A_{n}^{c}\right)$ and $\beta_{n}=p_{1}^{n}\left(A_{n}\right)$. We define $\beta_{n}^{\varepsilon }=\underset{\alpha_{n}<\varepsilon }{\min}\beta_{n}^{},0<\varepsilon <\frac{1}{2}$. Then:

(10) $$\underset{\varepsilon \to \infty }{\lim}\underset{n\to \infty }{\lim}\frac{1}{n}\log\beta_{n}^{\varepsilon }=-D\left(p_{0}∥p_{1}\right)$$

Target detection in radar signal processing is a binary hypothesis testing problem, which can be expressed as:

(11) $$\left\{ \begin{array}{l} H_{0}:Y_{i}=N_{i},\;\mathrm{no}\;\mathrm{target}\\ H_{1}:Y_{i}=X_{i}H_{i}+N,\;\mathrm{target}\;\mathrm{exists} \end{array}\right.$$

where i stand for the parameter for a particular round of radar signal adaptation at time i. Denote the RE by $D\left(p_{0}\left(Y_{i}\right)∥p_{1}\left(Y_{i}\right)\right)$, where $p_{0}\left(Y_{i}\right)$ and $p_{1}\left(Y_{i}\right)$ are the probability distribution functions (pdfs) $Y_{i}$ between two distributions $p_{0}$ and $p_{1}$ under the hypotheses $H_{0}$ and $H_{1}$, respectively.

From Stein’s lemma, it can be known that $\alpha_{n}$ is the false alarm probability and $\beta_{n}$ is the miss probability in the hypothesis testing problem. Then, if the false alarm probability $\alpha_{n}$ is fixed, the miss probability $\beta_{n}$ is exponentially small, with an exponential rate equal to the RE $D\left(p_{0}\left(Y_{i}\right)∥p_{1}\left(Y_{i}\right)\right)$. Therefore, in order to optimize detection performance, we should maximize the RE $D\left(p_{0}\left(Y_{i}\right)∥p_{1}\left(Y_{i}\right)\right)$. Under the power of the transmitted waveform constraint, the radar waveform optimization problem based on maximization of RE is expressed as:

(12) $$\begin{array}{l} \underset{X_{i}}{\max}D\left(p_{0}\left(Y_{i}\right)∥p_{1}\left(Y_{i}\right)\right)\\ s.t.\;\mathrm{tr}\left[X_{i}X_{i}^{H}\right]\leq P_{0} \end{array}$$

Then:

(13) $$\begin{array}{l} D\left(p_{0}\left(Y_{i}\right)∥p_{1}\left(Y_{i}\right)\right)=\int p_{0}\left(Y_{i}\right)\log\frac{p_{0}\left(Y_{i}\right)}{p_{1}\left(Y_{i}\right)}dY_{i}\\ =N\log\left[\det{\left(I_{K}+X_{i}R_{H}^{}X_{i}^{H}R_{N}^{-1}\right)}^{-1}\right]\\ +Ntr\left[\det{\left(I_{K}+X_{i}R_{H}^{}X_{i}^{H}R_{N}^{-1}\right)}^{-1}-I_{K}\right] \end{array}$$

Substituting (13) into (12), the radar waveform optimization problem can be formulated as:

(14) $$\begin{array}{l} \underset{X}{\max}\log\left[\det{\left(I_{K}+X_{i}R_{H}^{}X_{i}^{H}R_{N}^{-1}\right)}^{-1}\right]+tr\left[\det{\left(I_{K}+X_{i}R_{H}^{}X_{i}^{H}R_{N}^{-1}\right)}^{-1}-I_{K}\right]\\ s.t.\;\mathrm{tr}\left[X_{i}X_{i}^{H}\right]\leq P_{0} \end{array}$$

According to the [38], we can know that $X_{i}R_{H}X_{i}^{H}$ and $R_{N}^{}$ are both positive semi-definite Hermitian matrices. Let the eigen-decomposition of $X_{i}R_{H}^{}X_{i}^{H}$ and $R_{N}^{}$ be $U_{i}^{}\sum_{H}U_{i}^{H}$ and $V_{i}^{}\sum_{N}V_{i}^{H}$, where $\sum_{H}=diag\left(\left[\delta_{H,1},\delta_{H,2},\ldots ,\delta_{H,K}\right]\right)$ and $\sum_{N}=diag\left(\left[\delta_{N,1},\delta_{N,2},\ldots ,\delta_{N,K}\right]\right)$ and rank $\left(X_{i}R_{H}^{}X_{i}^{H}\right)\leq M$. So we have ${\hat{U}}_{i}^{}X_{i}R_{H}^{}X_{i}^{H}{\hat{U}}_{i}^{H}=\hat{\sum }$. Let ${\hat{X}}_{i}^{}={\hat{U}}_{i}^{H}X_{i}^{}$. The radar waveform optimization problem (14) can be reformulated as:

(15) $$\begin{array}{l} \underset{X}{\max}\log\left[\det{\left(I_{K}+R_{N}^{-1/2}{\hat{U}}_{i}^{}{\hat{X}}_{i}^{}R_{H}^{}{\hat{X}}_{i}^{H}{\hat{U}}_{i}^{H}R_{N}^{-1/2}\right)}^{-1}\right]+tr\left[{\left(I_{K}+R_{N}^{-1/2}{\hat{U}}_{i}^{}{\hat{X}}_{i}^{}R_{H}^{}{\hat{X}}_{i}^{H}{\hat{U}}_{i}^{H}R_{N}^{-1/2}\right)}^{-1}\right]\\ s.t.\;\mathrm{tr}\left[X_{i}^{}X_{i}^{H}\right]\leq P_{0}\\ \;{\hat{X}}_{i}^{}R_{H}^{}{\hat{X}}_{i}^{H}=\hat{\Sigma } \end{array}$$

From [38], we know that $\log\left[\det{\left(I+R\right)}^{-1}\right]+tr\left[{\left(I+R\right)}^{-1}\right]$ is a monotonic increasing function of positive semi-definite matrix R. Based on maximizing mutual information, the optimal solution $X_{i}^{opt}$ of (15) in this situation is given by:

(16) $$X_{i}^{opt}={\hat{U}}_{i}^{}{\left[\begin{array}{cc} 0_{\left(K-M\right)\times M} & {\hat{\Sigma }}^{1/2} \end{array}\right]}^{T}V_{i}^{H}$$

We design orthogonal waveforms from the Hadamard matrix, and modulate the power of the waveforms across the transmit antenna elements based on the maximization of RE criterion.

Step 2: Waveform Selection. The basic idea of the MI minimization scheme is that the optimal transmitted waveform is selected for the next time instant based upon the current target echo.

### 4.2. Parameter Estimation

We assume that the radar receiver has perfect knowledge of the transmitted waveform at all instants of time. Therefore, the information can be used to estimate parameters like the covariance matrices of the target response $R_{H_{i}}$ and the noise $R_{N}$:

(17) $$\begin{array}{l} R_{Y_{i}}=E\left(Y_{i}^{H}Y_{i}\right)=X_{i}^{H}R_{H_{i}}X_{i}+R_{N}\\ R_{Y_{i+1}}=E\left(Y_{i+1}^{H}Y_{i+1}\right)=X_{i+1}^{H}R_{H_{i}}X_{i+1}R_{N} \end{array}$$

where $R_{Y_{i}}$ and $R_{Y_{i+1}}$ are the variances of the received signals at time i and i+1. Solving the above Equation (17), $R_{H_{i}}$ and $R_{N}$ can be estimated. Two values will be used to generate the estimate of $Y_{i+2}$ for all values of $X_{i+2}\in \mathbb{C}$ using (8), where ℂ is the ensemble of the transmitted waveforms. $X_{i+2}\in \mathbb{C}$ will be selected based on MI minimization scheme. $R_{H_{i}}$ and $R_{N}$ are estimated at every instance of reception of $Y_{i}$, and the values is updated and used to generate new estimates for $Y_{i+1}$.

### 4.3. MI Minimization

We denote the MI between two random matrices $Y_{i}$ and $Y_{j}$ as $I\left(Y_{i},Y_{j}\right)$. If $Y_{i}$ and $Y_{j}$ are statistically dependent, $I\left(Y_{i},Y_{j}\right)$ is high. Therefore, if the MI between the current received signal and the estimated signal in the next time instant ($Y_{i}$ and $Y_{i+1}$) are statistically dependent, we cannot acquire significant gain in feature information of target. We intend to obtain uncorrelated and independent data samples from the radar scene to acquire more target feature information from scan to scan. Subsequently, we select those waveforms for emission that produce less statistically dependent received signals from the same target scene. That is to say, we desire to obtain the optimal waveforms by choosing from the ensemble ℂ a waveform that would minimize $I\left(Y_{i},Y_{i+1}\right)$. The MI between the received radar echoes at time i and i+1 can be expressed as:

(18) $$I\left(Y_{i},Y_{i+1}\right)=I\left(Y_{i}|X_{i}\right)+I\left(Y_{i+1}|X_{i+1}\right)-I\left(Y_{i},Y_{i+1}|X_{i},X_{i+1}\right)$$

where $I\left(Y_{i}|X_{i}\right)$ (or $I\left(Y_{i+1}|X_{i+1}\right)$) denotes the entropy of the received signals $Y_{i}$ (or $Y_{i+1}$) at time i (or i+1) given the knowledge of the transmitted signals $X_{i}$ (or $X_{i+1}$). According the definition of entropy, it is the measure of uncertainty. The term $I\left(Y_{i},Y_{i+1}|X_{i},X_{i+1}\right)$ in (18) are defined similarly. Let $p\left(Y_{i}|X_{i}\right)$ (or $p\left(Y_{i+1}|X_{i+1}\right)$) be the conditional pdf of $Y_{i}$ (or $Y_{i+1}$) given $X_{i}$ (or $X_{i+1}$). According to the definition of entropy, we obtain:

(19) $$\begin{array}{l} I\left(Y_{i}|X_{i}\right)=\int -p\left(Y_{i}|X_{i}\right)In\left[p\left(Y_{i}|X_{i}\right)\right]dY_{i}\\ =NKIn\left(\pi \right)+NK+NIn\left[\det\left(X_{i}^{H}R_{H_{i}}X_{i}+R_{N}\right)\right] \end{array}$$

(20) $$\begin{array}{l} I\left(Y_{i+1}|X_{i+1}\right)=\int -p\left(Y_{i+1}|X_{i+1}\right)In\left[p\left(Y_{i+1}|X_{i+1}\right)\right]dY_{i+1}\\ =NKIn\left(\pi \right)+NK+NIn\left[\det\left(X_{i+1}^{H}R_{H_{i+1}}X_{i+1}+R_{N}\right)\right] \end{array}$$

and:

(21) $$\begin{array}{l} I\left(Y_{i},Y_{i+1}|X_{i},X_{i+1}\right)\\ =2NKIn\left(\pi \right)+2NK\\ +NIn\left[\det\left(X_{i}^{H}R_{H_{i}}X_{i}+R_{N}\right)\right]\\ +NIn\left[\det\left(X_{i+1}^{H}R_{H_{i+1}}X_{i+1}+R_{N}\right)\right]\\ +NIn\left[\det\left(I_{\left(M\times M\right)}-D_{i,i+1}^{2}\right)\right] \end{array}$$

where $I_{\left(M\times M\right)}$ is the identity matrix, $D_{i,i+1}^{}$ is the diagonal matrix, which is acquired by singular value decomposition (SVD) of the covariance matrix. The covariance matrix can be expressed as:

(22) $$R_{Y_{i},Y_{i+1}}=E\left\{Y_{i}^{H}Y_{i+1}\right\}=X_{i}^{H}R_{H_{i}}^{}X_{i+1}$$

By solving above Equations (19)–(21), MI between the received radar echoes at time i and i+1 can be obtained as follows:

(23) $$\begin{array}{l} I\left(Y_{i},Y_{i+1}\right)=-NIn\left(\det\left(I_{\left(M\times M\right)}-D_{i,i+1}^{2}\right)\right)\\ =-N\sum_{m=1}^{M}In\left(1-{\left(d_{i,i+1}^{m}\right)}^{2}\right) \end{array}$$

where $d_{i,i+1}^{m}(d_{i,i+1}^{1}\geq d_{i,i+1}^{2}\geq \ldots \geq d_{i,i+1}^{M})$ are the diagonal elements of $D_{i,i+1}^{}$. Finally, we form the minimization MI problem as follows:

(24) $$\begin{array}{ll} MI_{\min}= & \underset{X_{i+1}\in {\mathbb{C}}_{X_{i}}}{\min}\left\{-N\sum_{m=1}^{M}In\left\{1-{\left(d_{i,i+1}^{m}\right)}^{2}\right\}\right\}\\ & s.t.\;\mathrm{tr}\left[X_{i+1}^{H}X_{i+1}\right]\leq P_{0} \end{array}$$

where $P_{0}$ is transmitted power. From [37], the optimization problem (24) is solved by choosing $X_{i+1}\in {\mathbb{C}}_{X_{i}}$.

Step 1 designs the optimization waveform ensemble with the purpose of maximizing RE over the spatial domain, and step 2 chooses the sequence for each transmit antenna element from the optimization waveform ensemble with an objective of minimizing MI over the temporal domain. The information embedding MIMO waveform optimization process can be described in Algorithm 1.

**Algorithm 1.** The information embedding MIMO waveform optimization algorithm**Step 1:** Initializing iteration index i=0, the covariance matrix $R_{H_{0}}$ and $R_{N_{0}}$. **Step 2:** At time i=0, solve for the ensemble of transmitted waveforms ${\mathbb{C}}_{X_{0}}$ based on maximization RE criterion over the spatial domain as presented in step 1. **Step 3:** At time i=1, Form an estimate of the received signal $Y_{1}$, based on the current estimate for TIR by using (3). The received signals are used to extract the TIR. **Step 4:** At time i=1, solve for $X_{1}$ transmitted waveforms based on the minimization MI criterion over the temporal domain as presented in step 2. **Step 5:** At time i=1, emission $X_{1}$ and the updated $R_{H_{1}}$ and $R_{N_{1}}$ by using the current received signal $Y_{1}$. **Step 6:** If $i=I_{\max}$, the process ends; otherwise, we need to go back to Step 2 and repeat.

The proposed information embedding strategy can be summarized as follows:

(1) The MIMO radar waveform with MPPSK embedding symbols is transmitted. The communication link is between any two or more different nodes.
(2) The proposed radar-communication transceiver updates the estimate of TIR and utilizes this information to choose the optimal waveform for transmission. An adaptive feedback loop enables the delivery of the TIR information to the transmitter.
(3) The proposed system adapts its MPPSK modulated inter-pulse duration and adjusts its transmitted waveform to suit the time-varying environment.
(4) The received signals are processed by matched filters, which demodulate the MPPSK signal and convert them into the corresponding M-ary sequence. The received signals are also used to extract the TIR.

The proposed architecture is similar to cognitive MIMO radar, where the systems adopt a constant learning approach by updating the target parameters.

## 5. Simulation Results

We set orthogonal sequences of the Hadamard matrix over the transmit antenna elements. As described in the previous sections, the orthogonality between the proposed systems is maintained for radar waveform optimization purposes. Next, the received signals are matched filtered to estimate the propagation delay at the receivers. The communication data are demodulated and the radar signal processing is carried out, separately. In this way, we can obtain an acceptable SER for communications. The MSE matrix of TIR is estimated in the subsequent pulse interval. The transmitted signals are later selected by the waveform optimization module as shown in Figure 1.

We assume that the amplitudes of the received signals vary independently from scan to scan (Swerling case III). The random RSC $a={\left|h_{m,n}\right|}^{2}$, a≥0 is exponentially distributed, which can be denoted as:

(25) $$f\left(a\right)=\frac{1}{a_{av}}\exp\left(-\frac{a}{a_{av}}\right)$$

where $a_{av}$ is the average variance of RCS fluctuation. The simulation parameters are shown in Table 1.

### 5.1. Target Detection Performance

**Figure 4:**
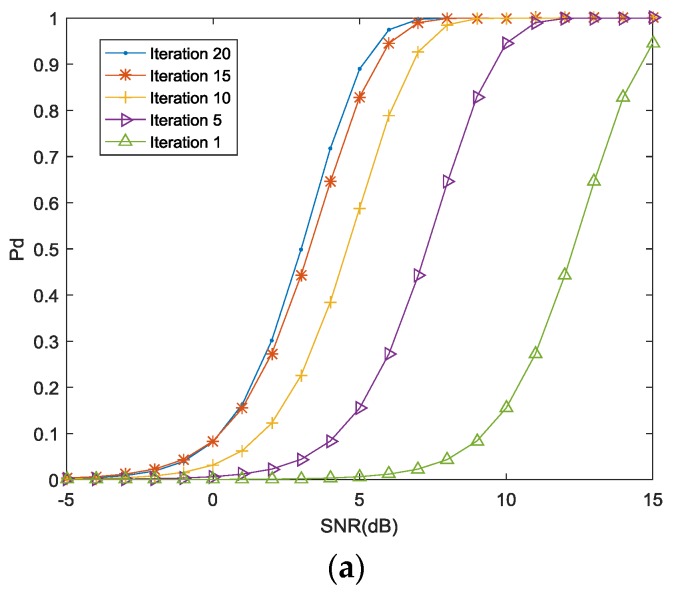

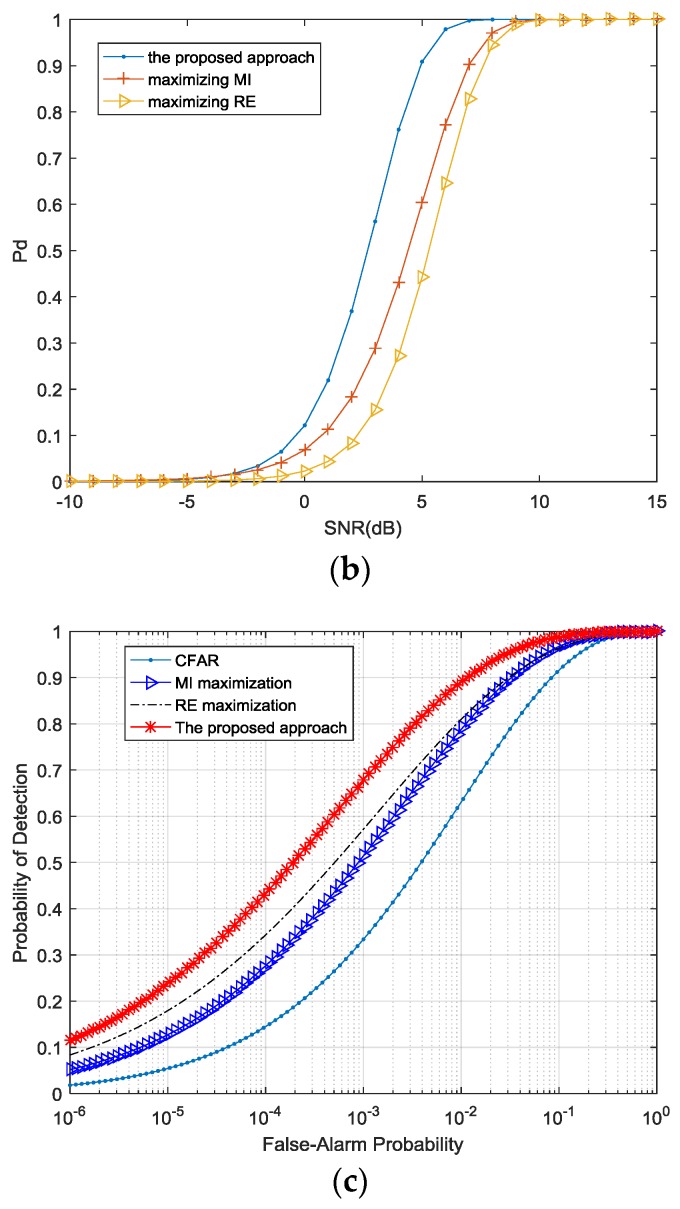
(**a**) Detection probability for various iterations of the proposed approach; (**b**) Detection probability for three approaches; (**c**) The ROC for four configurations.

Figure 4a indicates the detection probability achieved by the proposed approach for false alarm probability $p_{fa}=5\%$. For a stationary radar scene, 1000 simulations have been run for each at a particular value of the received SNR. The next transmission waveform is selected according to the proposed two-step optimization algorithm, and the optimization process is repeated for 20 iterations. As seen from Figure 4a, the required SNR value decreases as the number of iterations increase for a fixed detection probability. The proposed algorithm converges after 15 iterations, yielding a detection probability of 0.9 at SNR = 4 dB as compared to SNR = 14 dB at the first iteration. However, the detection probability does not show further improvement after 20 iterations.

In Figure 4b, we compare the detection probability for optimization waveforms selected by the proposed scheme to the probability for waveform based on MI maximization, and also compare this result with the optimized waveform based the RE maximization as presented in [29].

As the proposed algorithm utilizes the RE and MI during the scans interval, the MIMO radar-communication transceiver adapts its radar signal better than waveform based on MI maximization to the fluctuating target RCS in multipath environments. On the other hand, optimized waveform based on the RE is unable to match the time-varying TIR after multiple iterations. Hence, the detection probability is suboptimal in this case.

Finally, Figure 4c shows the ROC for four different configurations. (1) constant false alarm rate (CFAR) detection based on Neyman-Pearson criterion using the phased antenna arrays; (2) 4×4 MIMO radar employing the maximization of MI as presented in [39]; (3) 4×4 MIMO radar employing the maximization of the RE as presented in [39]; (4) 4×4 MIMO radar-communication transceiver employing the proposed waveform optimization scheme.

The curves for the RE maximization, the MI maximization and the proposed waveform optimization algorithms are generated at the end of 20 iterations. For a false alarm probability $p_{fa}=0.01$, the target detection probability generated by the proposed algorithm is approximately 0.9 as compared with 0.8 offered by the MI maximization approach, 0.8 by the relative entropy maximization approach, and 0.6 by the Neyman-Pearson criterion. No significant improvement is observed after 20 iterations.

### 5.2. TIR Estimation Performance

**Figure 5:**
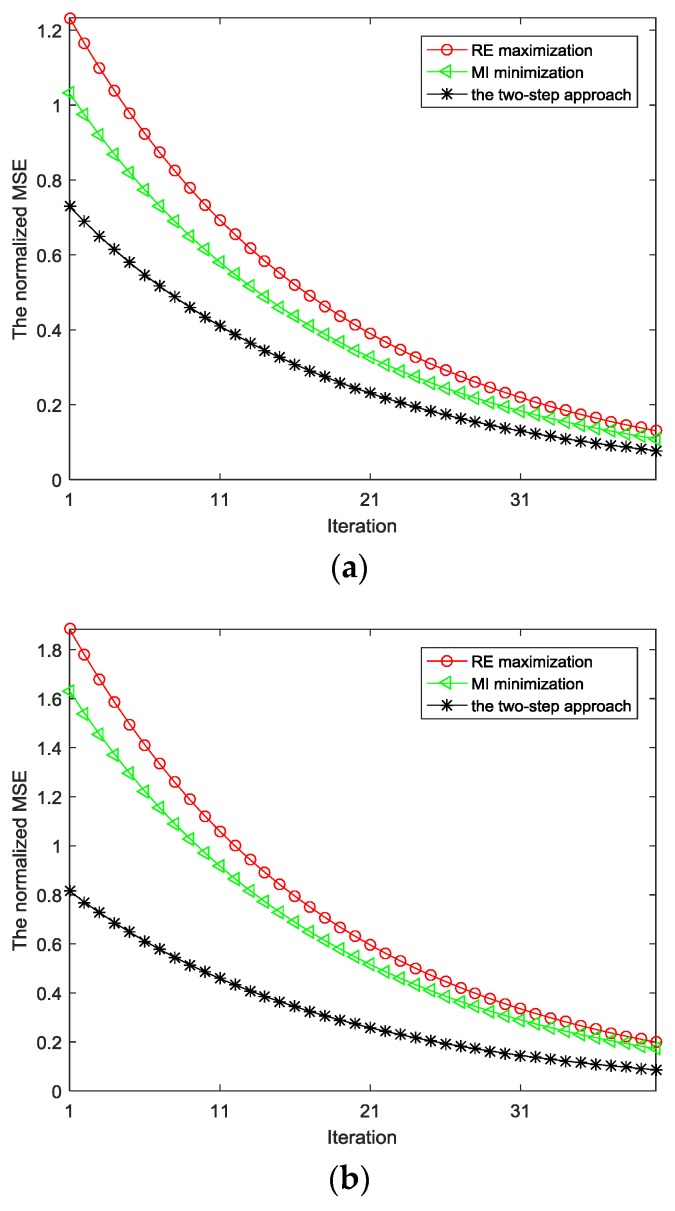
(**a**) The MSE of TIR estimation under power constraint; (**b**) The MSE of TIR estimation under power, PAPR constraint.

Figure 5a indicates the normalized MSE with regard to the estimation of TIR under the constraint of transmitted power in multipath environments. This plot demonstrates an improved MSE performance for the proposed optimization approach as compared with the maximization of RE and minimization of MI modules. As seen from Figure 5a, the normalized MSE of TIR based on the proposed algorithm is smaller than that using the maximization of RE. Similarly, the normalized MSE of TIR based on the proposed algorithm is smaller than that using the minimization of MI, particularly for the first few iteration.

Figure 5b indicates the MSE with regard to the estimation of TIR under the constraint of transmitted power, PAPR in multipath environments. The normalized MSE of TIR based on the proposed scheme and two individual approaches are compared to verify the efficiency of the proposed scheme at each iteration step.

### 5.3. Communication SER

**Figure 6:**
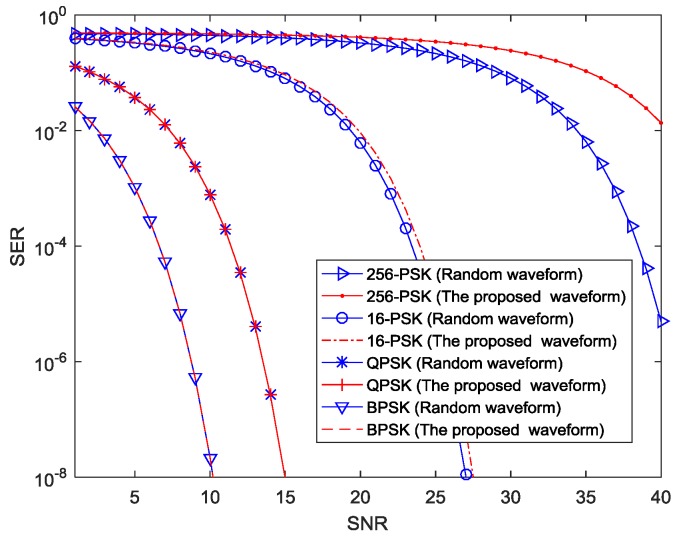
Comparative SER results of BPSK, QPSK, 16-PSK, 256-PSK.

We generate two types of random waveforms and the proposed optimization waveforms as presented in Section 4. 4×4 MIMO radar-communication transceiver is employed. Then, we discuss the SER performance of the proposed information embedding scheme using BPSK, QPSK, 16-PSK, and 256-PSK constellations. To test the SER performance, a total number of $16\times 10^{7}$ random symbols are used. These signals corresponds to data rate of R = 1.2, 2.4, 4.8, and 9.6 Mbps, respectively. Figure 6 illustrates the SER versus SNR for all constellation sizes.

As seen from Figure 6, the SNR performance of BPSK signal may be improved by approximately 5 dB, 16 dB and 33 dB as compared with QPSK, 16-PSK, 256-PSK, respectively. The figure shows that the smaller the constellation size is, the better the SER performance will be.

Figure 6 also shows that for BPSK, QPSK, and 16-PSK constellations, the SER performance of optimized waveforms is nearly the same as that of the random waveforms. However, for the 256-PSK constellation, the SER performance of the proposed waveforms is worse than random waveforms. As constellation size is increased, the cross-correlation levels become higher. Therefore, we choose reasonable constellation size results in a tradeoff between SER and data rate requirements from a communications perspective.

## 6. Conclusions

In this paper, a novel waveform design concept for an adaptive distributed MIMO radar-communication system has been studied that allows for simultaneous wireless communications and radar operation. A novel approach for embedding communication data into MIMO radar signals using MPPSK waveforms is presented. The new waveform optimization approach is addressed for providing high performance gains in terms of TIR estimation and probability of target detection. The proposed method also facilitates high data rate performance for the communications application. The discussed waveform design concepts offer interesting perspectives for the realization of future sensor devices in intelligent transportation systems. Nevertheless, there is an increase in the computational load due to two steps in the waveform optimization. Future works will look into the tradeoff between the performance enhancement and the computational complexity involved. Additionally, it could be interesting to analyze the design of information embedding MIMO waveforms for tracking applications.

## Figures and Tables

**Table 1:** Simulation Parameters.

Simulation Parameters		
$E_{s}$	Transmitted power	1
B	Bandwidth	500 MHz
L	Length of signal	30
$f_{0}$	PRF	100 KHz
$p_{fa}$	False alarm probability	0.02
$p_{d}$	detection probability	0.95
PAPR	peak-to-average ratio	3 dB
$f_{s}$	the sampling frequency	500 MHz
$f_{c}$	carrier frequency	8 GHz
